# A Multilayer Architecture towards the Development and Distribution of Multimodal Interface Applications on the Edge

**DOI:** 10.3390/s24165199

**Published:** 2024-08-11

**Authors:** Nikolaos Malamas, Konstantinos Panayiotou, Apostolia Karabatea, Emmanouil Tsardoulias, Andreas L. Symeonidis

**Affiliations:** 1 Faculty of Engineering, Aristotle University of Thessaloniki, 541 24 Thessaloniki, Greece; klpanagi@ece.auth.gr (K.P.); etsardou@ece.auth.gr (E.T.); symeonid@ece.auth.gr (A.L.S.); 2 Gnomon Informatics S.A., 570 01 Thessaloniki, Greece; a.karabatea@gnomon.com.gr

**Keywords:** smart assistants, cyber–physical systems, smart environments, internet of things, human–computer interaction, application development

## Abstract

Today, Smart Assistants (SAs) are supported by significantly improved Natural Language Processing (NLP) and Natural Language Understanding (NLU) engines as well as AI-enabled decision support, enabling efficient information communication, easy appliance/device control, and seamless access to entertainment services, among others. In fact, an increasing number of modern households are being equipped with SAs, which promise to enhance user experience in the context of smart environments through verbal interaction. Currently, the market in SAs is dominated by products manufactured by technology giants that provide well designed off-the-shelf solutions. However, their simple setup and ease of use come with trade-offs, as these SAs abide by proprietary and/or closed-source architectures and offer limited functionality. Their enforced vendor lock-in does not provide (power) users with the ability to build custom conversational applications through their SAs. On the other hand, employing an open-source approach for building and deploying an SA (which comes with a significant overhead) necessitates expertise in multiple domains and fluency in the multimodal technologies used to build the envisioned applications. In this context, this paper proposes a methodology for developing and deploying conversational applications on the edge on top of an open-source software and hardware infrastructure via a multilayer architecture that simplifies low-level complexity and reduces learning overhead. The proposed approach facilitates the rapid development of applications by third-party developers, thereby enabling the establishment of a marketplace of customized applications aimed at the smart assisted living domain, among others. The supporting framework supports application developers, device owners, and ecosystem administrators in building, testing, uploading, and deploying applications, remotely controlling devices, and monitoring device performance. A demonstration of this methodology is presented and discussed focusing on health and assisted living applications for the elderly.

## 1. Introduction

The field of Natural Language Processing (NLP) has made significant advancements in recent years, leading to the development of efficient Smart Assistants (SAs). Around 35% of US adults are already using SAs daily, and it is expected that almost half of US adults will own and use such assistants by 2025 [1]. This trend started with the release of SAs by major tech companies, such as Google Home and Amazon Alexa, even though the smart “landscape” was not yet fully in place. Today, commercial SAs are robust and easy to set up, connect, and use. They follow a cloud-native approach to deliver functionality and realize improved understanding and responsiveness, being able to engage in contextual conversations and assist users with everyday tasks. Apart from tech-savvy users, these SAs find a market in people who live alone and require assistance or even companionship, such as the elderly.

Despite their popularity, these SAs face criticism that can be summarised in three categories: (a) closed products that drive users into vendor lock-in, (b) limited personalization and feature management, and (c) inability to build custom applications. These devices only operate with certified devices for full functionality or linked devices for basic functionality. The closed SA ecosystem may prohibit even advanced users from developing custom applications and “tampering” with the SA engine. For example, it is extremely hard to allow the execution of specific functionality for predefined user groups, such as parental control, or to limit financial transaction functionality to a particular user or group of users. Additionally, most commercial SAs support only widely-spoken languages, directly contradicting several studies that have addressed the need for multilingualism [2], especially for less frequently spoken languages. Customization and personalization for different user groups, including non-native English speakers and the elderly, are also important for increasing the impact of SAs.

The fact that SAs allow for the control of multiple devices and enhance usability via vocal, haptic, and multimodal interfaces is pivotal in light of the increasing number of IoT devices. Research studies show that the number of connected devices in 2020 was 18 billion [3], while this number is expected to exceed 25 billion by 2030 [4]. Similarly, more than 125 million people worldwide frequently use a voice-based assistant [5]; in 2022 there were around 140 million SAs in the US alone [6], and half of the adult population is expected to use such systems in the next years [1]. This increase is driving the need to develop more sophisticated applications that control more heterogeneous devices while embedding intelligence and complex behaviors. Thus, a methodology that allows for more flexible application development for SAs is deemed necessary.

The current work presents a methodology and framework that support the design, development, and distribution of SA applications along with a multi-layer SA architecture that allows for remote distribution, management, and monitoring of SA applications. The proposed application development methodology is capable of offloading low-level device-specific details, and provides a device and a cloud API that applications can use to access on-device functionality (e.g., speech, music playback, and touchscreen input/output) and remote services (e.g., weather forecasting and geospatial information). It also includes a modular and fully transparent architecture that allows for remote management (installation, uninstalling, execution, etc.) of applications from a centralized cloud-based platform while applications are processed and executed on the edge, thereby protecting sensitive user data. This approach provides a clear and open process for developing and deploying multimodal applications for various domains and custom use cases. Our methodology was applied as part of the SITIES project (https://sities-project.gr/en/, accessed on 30 July 2024) (“Support System for the Elderly with Smart Devices”), resulting in the creation of an SA named ELSA focusing on home care and eHealth-related scenarios for the elderly.

The rest of this paper is structured as follows: Section 1.1 discusses the latest advances and related work regarding concerns about commercial SAs and Edge SA-engineered solutions; Section 2 provides a detailed discussion of the requirements and specifications of the cloud and edge layers; Section 3 focuses on the demonstration conducted as part of this study to assess the proposed multilayer framework in an end-to-end manner; finally, Section 4 summarizes our study and explores future extensions of the current work.

### 1.1. Related Work

Commercial SAs are becoming increasingly popular as a more intuitive and convenient interface for interacting with computer systems while also augmenting human capabilities in the home [7]. Major players such as Amazon, Google, Microsoft, and Apple have developed SAs to provide personalized assistance to users in performing everyday tasks such as scheduling appointments, setting alarms, playing music, and controlling smart home devices. These systems can easily connect to their ecosystems and compatible devices/sensors, creating intelligent environments that can follow users’ requests and provide a variety of services. This is achieved through the use of various technologies, including Natural Language Processing (NLP) and Artificial Intelligence (AI), which help to understand user input and provide appropriate responses and actions. Most of these SAs also offer specialized frameworks, such as Alexa Skills Kit (https://developer.amazon.com/en-US/alexa/alexa-skills-kit, accessed on 30 July 2024) and Google Home Device SDK (https://developers.home.google.com/device-sdk, accessed on 30 July 2024), for developing custom applications that can be installed on their devices and vendor ecosystem. However, when selecting such systems it is important to consider several concerns.

First of all, it is common practice for companies to build devices that are fully compatible with their official products, leading to a vendor lock-in ecosystem in which switching to another product or company becomes difficult, time consuming, and costly, as discussed in [8]. Transparency is another important issue in conversational systems [9,10,11,12], especially for older adults [13]. In several heterogeneous studies, recommender [14] and customer support [15] systems have addressed this aspect to develop approaches that can increase users’ trust and confidence, leading to an improved overall experience [16]. This is important because recent surveys showed that only around 50% of people trust SAs [9]. In addition, 70% of users who experience SA problems choose to abandon the assistant rather than try to rephrase their queries in the hope of getting better results [17]. However, allowing users to choose the available dialogue applications in their assistants while thoroughly presenting their trigger phrases and functionality is a much more convenient and transparent approach, and is the one that we implement here.

In addition to user satisfaction and engagement, trust can also be achieved by personalizing SA functionality [18]. Personalization can be described as offering the right services to each user according to their specific needs. Commercial SAs usually achieve this by processing user habits and offering targeted recommendations or services, in some cases by allowing users to create their own routines; however, this raises privacy concerns, as discussed above, [19]. As these are general purpose systems, they usually do not have functionalities that are specific to certain user groups or domains. For example, user groups such as the elderly, non-English speakers, and even parents have similar needs among their group members. The presented methodology has been designed in an way that allows developers to design and implement such domain- and user-specific applications to select the exact ones that their device will handle according to their needs, such as non-English support or eHealth scenarios focused on elderly people living alone, as demonstrated in the SITIES project.

Furthermore, transparency and trust issues can be efficiently addressed by embedding some or all SA functionality on edge devices, as has been discussed in a handful of studies. In particular, a lightweight SA hosted on a Raspberry Pi 3 was presented in [20]. The proposal follows a modular and extensible design integrated into a smart home environment while providing a combined vocal and visual interface. Similarly, the task-oriented SA presented in [21] works entirely on the edge without requiring an internet connection. In this way, all interaction remains in the local domain, increasing the privacy of end users. SimplyRetrieve [22] is another conversational system for retrieving information from local knowledge bases. However, these solutions cannot be considered end-to-end systems that allow for the management, development, and distribution of multimodal conversational applications.

Overall, edge computing results in increased security and privacy at the cost of higher response times [23]. Such devices can communicate and exchange data or request specific actions effectively at high speeds via several different protocols, including REST interfaces as well as more messaging-oriented protocols such as MQTT [24], AMQP [25], and Redis (https://redis.io/, accessed on 30 July 2024), as presented in several previous studies [26,27,28,29]. These devices can process and analyze multimodal data such as images, videos, and text in smart environments [30], leading to more complex systems in which assistants can offer a more natural and engaging user experience [31]. For example, in one study focusing on assisting people with disabilities and bed-bound patients [29], IoT devices were connected with service robots and handled via a multimodal interface in which users could interact through speech, touch, and gestures to control the robots and devices in the room. In another study, a multimodal SA was integrated into a smart home environment to manage existing smart devices through vocal, textual, and haptic interfaces [32]. In addition, a multimodal SA framework was presented in [33] with the aim of assisting elderly people by offering personal recognition during interactions, object detection of the nearby area, and query-based assistance. Following the state of the art, our proposed architecture also allows developers to build multimodal applications, particularly vocal and haptic ones, that can connect with custom IoT devices and manage a complete smart environment, ultimately providing a better overall user experience.

Finally, regarding software selection, there are several open-source frameworks for task-based SA development, such as wit.ai [34], Botpress [35], Rhasspy [36], and Rasa [37]. Among these, the latter is considered to be one of the most powerful frameworks, as it achieves similar NLP performance to commercial task-based frameworks such as Amazon Lex, Google Dialogueflow, and IBM Watson [38,39] while being more scalable, fully customizable, and self-hosted, as discussed in [8]. In addition, it can provide localized wizards, as it can support any language given the appropriate data, as demonstrated in several languages including Spanish [40], Vietnamese [41], and Greek [2]. However, it should be noted that these frameworks focus only on the development of conversational scenarios, and cannot be considered end-to-end solutions that can be independently deployed in a household. Hence, a larger, modular, and more extensible approach is required to produce complete SA systems and applications.

The novelty of the current work resides in the proposal of a multilayered architecture based on open-source hardware and software to address the aforementioned drawbacks of existing SAs. This architecture aims to reduce complexity while increasing productivity in the development and distribution of applications with multimodal user interfaces for SA devices on the edge. Specifically, our approach **avoids vendor lock-in**, as all software modules are open for utilization/management/enhancement and developed applications follow only a few rules for their integration with the app management system, allowing for maximum flexibility regarding their offered functionality. These applications are developed in Python programming language, and developers can utilize the SA API, through which **vocal and haptic interfaces** are offered. Furthermore, developers can easily **connect to third-party services** that reside on the cloud, as well as to **custom devices** on the cloud or in the local smart environment, making the respective applications richer in content and more useful, ultimately resulting in a more open and flexible approach. On the contrary, developing and deploying applications in commercial SAs is rather cumbersome and restrictive to the vendor’s ecosystem when performed with their provided development kits, while the existing open-source SA frameworks are not end-to-end solutions for SA deployment, application distribution, and integration with other devices in a smart ecosystem. Regarding applications, they are deployed locally, as opposed to the remote deployment used in other SAs. Containers are used to provide **privacy and isolation**, ensuring that a developer of an application cannot access data existing in the device or data produced by other apps. Furthermore, the cloud platform shows the installed applications in real time as well as the verbal interactions of the SA, allowing for **transparency** regarding what the SA understands and what applications it is deploying. Regarding **personalization**, most current systems can be considered general purpose, offering limited localization and customization features while being unable to adapt efficiently to specific user groups. In our architecture, the verbal interface language is easy to change, as we rely on open-source tools for NLP and NLU. Additionally, developers can ask for personalized information from the user when the application is installed, allowing the application to properly offer services for the specific user (e.g., a weather forecast application may require the provision of an address or city name). All of these aspects—transparency, privacy, isolation, and personalization—are crucial, as their absence limits the user’s internal knowledge and full ownership of the device they are using and of the data being collected and processed. This becomes particularly important when users are much more vulnerable, such as when sensitive data are being transmitted, or when users are less familiar with technology, as is often the case for elderly individuals.

## 2. Implementation

This section discusses the proposed system architecture, multimodal interfaces for the SA, and application management methodology.

### 2.1. Architecture

**Figure 1:**
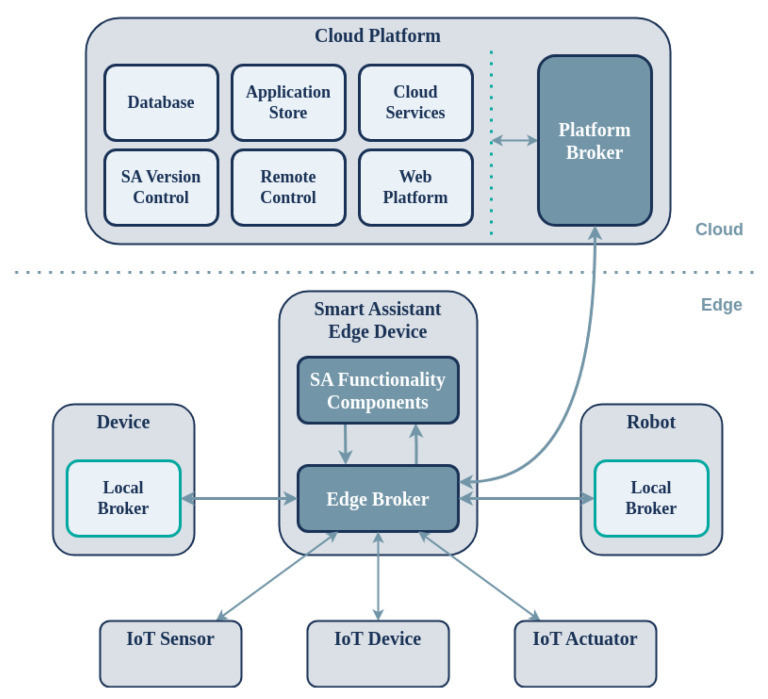
Architecture of the framework for supporting rapid development of applications for SAs on the edge.

The overall architecture comprises two distinct components, namely, the SAs deployed on the edge and a centralized platform on the cloud offering interconnection with the assistance of local and remote brokers (Figure 1). The proposed multilayer architecture shares common requirements with the IoT and CPS domains, including connectivity and communication between the edge and cloud components of the system, remote monitoring and management, application distribution and deployment, and device firmware updates.

#### 2.1.1. Cloud Platform

The cloud platform comprises four major subsystems: (a) the Web Platform; (b) the Application Store; (c) the Platform Broker; and (d) Cloud Services. The Web Platform provides a web interface for managing and monitoring SA devices on the edge, for example in a smart home environment. The Application Store connects to the Web Platform and allows developers to store, download, share, and distribute applications for their SAs. The Platform Broker acts as the communication and messaging middleware between the edge and cloud, connecting SA devices to the backend services of the platform. Finally, Cloud Services add remote functionality such as weather forecasts and calendar reminders to the applications via the CloudAPI (C-API) provided to the developers. The Cloud Platform also includes components that pertain to the integration of external data and services with the system (SaaS), including databases (DB), user management, a dataset search and annotation module, and a services search and annotation module.

All SA devices are supported by a centralized cloud platform that constantly communicates with each device and is responsible for three main processes: (a) software releases and distribution on the edge; (b) remote real-time monitoring of devices (telemetry); and (c) application management. The platform enables users to remotely manage their devices, including monitoring resource-level information such as CPU and RAM usage, remotely adjusting audio volume, upgrading on-device software components, and conveniently installing and managing applications from the Application Store. The SA facilitates the on-demand installation of heterogeneous applications by receiving, installing, and executing them dynamically. These applications can make use of web services that are hosted on the cloud, thereby offloading computationally intensive processes. The system is supported by a suitable programming and computing infrastructure, allowing independent developers and companies to create and manage new applications and services with ease.

Regarding application creation and management from the developers’ point of view, the Application Store is responsible for storing, analyzing, and distributing applications. Developers can store or delete an application while maintaining different versions to facilitate updates and compatibility. Applications are initially private, and only become available to users upon approval by their creator. The Application Store communicates with the database through a set of API interfaces. In addition, it provides services for directly testing/debugging the expected behavior of applications on an SA device in real time without requiring previous installation of the application.

**Figure 2:**
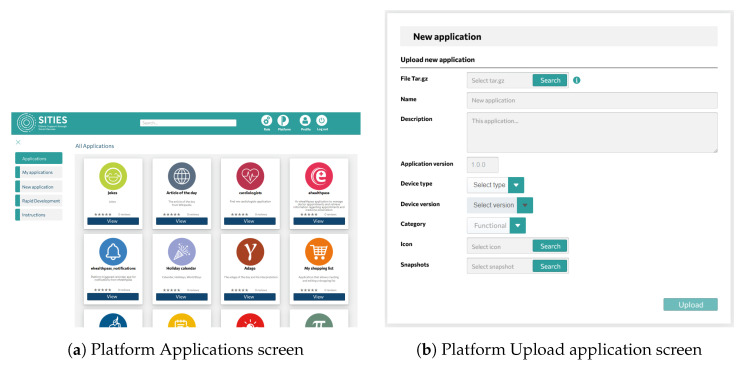

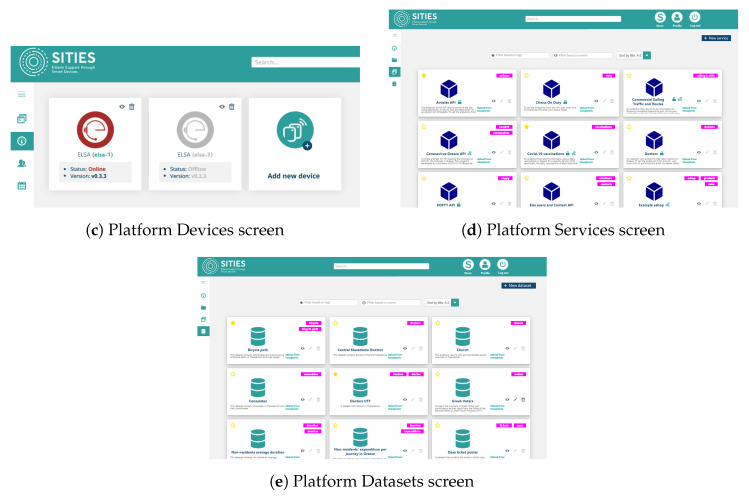
Platform screens showing the supported functionality.

From the point of view of device owners and the caregivers, the platform allows for device management, including software management, data monitoring related to device operation, and application management, as depicted in Figure 2. Specifically, the following high-level operations and functionality are provided in different platform screens:

- Each SA device periodically sends heartbeat messages, indicating the health status of each installed software module.
- Device monitoring provides information on device metrics such as computational load, CPU and memory load, and hard disk usage, which is reported by the Health Monitoring module running on each device on the edge.
- Device remote control allows for functions such as setting the volume of the SA device and performing start/restart/shutdown operations.
- Authorized users with permission to manage a device can remotely install, uninstall, start, or stop applications on the SA device on the edge.

Below, the proposed Smart Assistant device is presented along with an overview of its position within the overall architecture.

#### 2.1.2. The Smart Assistant Device

The proposed SA uses a Raspberry Pi 4 (RPI-4 ModelB 8 GB) as the central computation unit to connect sensors and actuators. The peripherals include a Raspberry Pi Camera Module V2 (8 MP, 1080p) for capturing images or video, a Pi Display 7" HDMI 800 × 480 Capacitive Touchscreen USB, a ReSpeaker Mic Array v2.0 for capturing sound, and a pair of stereo speakers. These peripherals are the physical resources of the device that applications can access to interact with the user and the environment.

**Figure 3:**
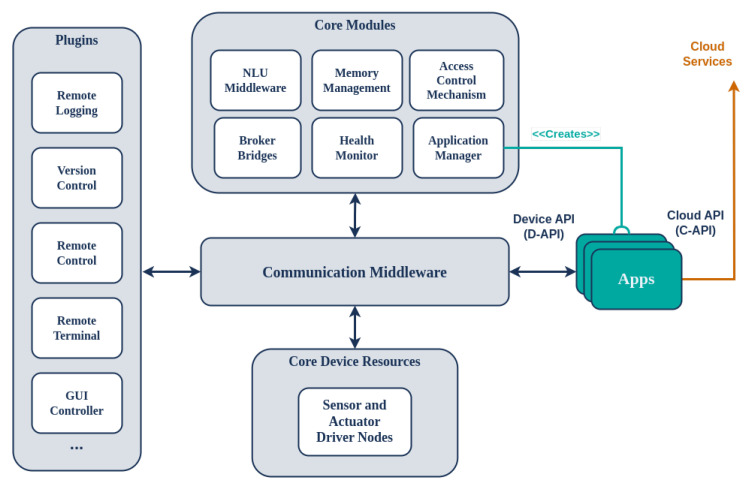
Software architecture of the SA device.

The software architecture of the device is presented in Figure 3, offering a plugin-based approach for on-device distributed components. These communicate via a common middleware to support connectivity, communication, and memory management, among other things, of the software components running on the SA device. Redis (https://redis.io/, accessed on 30 July 2024), an open source (BSD-licensed) in-memory data structure store, is used as the database, cache, and message broker software. To support proper communication with the cloud platform, the SA uses a protocol-agnostic communication middleware implemented using Commlib [42], an internal DSL in Python for broker-based communication and messaging. Commlib implements several communication patterns for endpoints via the message broker, such as PubSub, RPCs, and preemptive services with feedback. Commlib also provides communication between the on-device software components via the Redis broker and connectivity to the platform through an AMQP broker located on the cloud (RabbitMQ (https://www.rabbitmq.com/, accessed on 30 July 2024)). The middleware of the device comprises static software components and the applications that they run. The on-device software components communicate with the peripheral sensors and actuators via the *DeviceAPI (D-API)*, interfaces for communication with necessary services of the Application Store, and software for managing the applications (start/stop/pause/delete/installs). Each component locally communicates with APIs via the on-device communication middleware and remotely communicates with the platform via the Platform Broker (see Figure 1).

**Figure 4:**
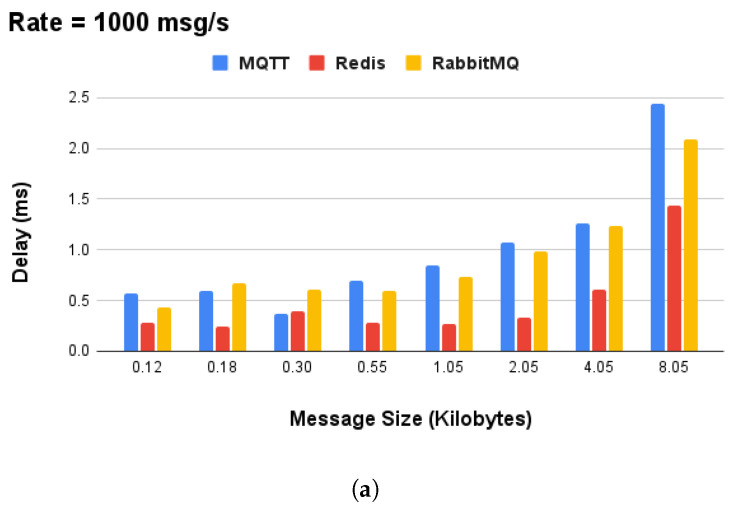

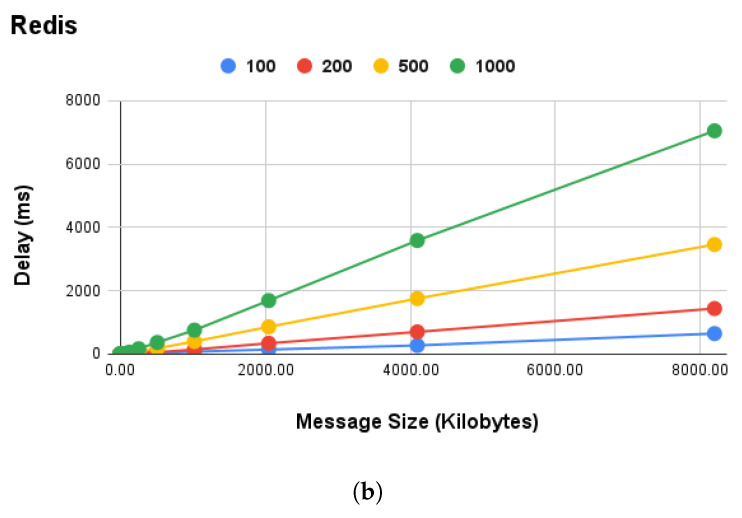
Performance analysis of commonly used message broker technologies for local communication on Raspberry Pi 4 devices: (**a**) performance comparison of MQTT, Redis, and AMQP in terms of delay and (**b**) performance of Redis broker for variable message rate and data size.

The selection of Redis technology for the on-device communication layer was not arbitrary, being based on performance experiments conducted on the Raspberry Pi RPI-4. Commlib supports the MQTT, Redis, and AMQP protocols, and provides an abstract protocol-agnostic API for asynchronous PubSub, synchronous RPC, and mixed-action (preemptive services with asynchronous feedback) communication patterns. Furthermore, we measured the data size (in kilobytes) of messages for the SA device’s local communication in order to focus on the area of interest, which is in the range of [0.12,8.05] kilobytes. The performance experiments were executed for a variable message size (in kilobytes) and message rate, that is, the number of messages per second transmitted over the message brokers, for 100,200,500, and 1000 msgs/s. In this sense, our results indicated Redis as the best overall broker technology to use for local communication. In terms of performance, it achieved an average message rate of 1000 msgs/s and message size in the range of [0.12,8.05], as shown in Figure 4.

The software that resides in the SA is divided into Core Modules and Plugins. The **Core Modules** are those that are essential for the basic operational functionality of the SA, managing applications, NLU, communicating with the Cloud Platform, monitoring health, and managing access control. Furthermore, the **Core Device Resources** are software drivers which control peripheral sensors and actuators; they provide the relevant interfaces to the rest of the system via the communication middleware, as presented in Figure 3. On the other hand, the **Plugins** are optional and add extra functionality to the SA, including remote logging (system and applications), remote version control, touch screen control, remote SSH tunnels, etc.

Finally, SA devices can act as a gateway for other devices along with individual IoT sensors and actuators. This broker-based design enables integration via established communication protocols such as MQTT (used as the Edge Broker in Figure 1). In this way, the SA forwards data from IoT sensors to applications as well as commands from applications to IoT actuators and other devices. Examples of such applications are everywhere in contemporary smart environments used for controlling remotely accessible sensory and actuator smart devices.

### 2.2. Multimodal User Interfaces

In the context of the current work, we have built an SA based on open-source software/hardware components along with a set of user requirements. A process of requirements elicitation took place before the actual design of the overall software/hardware architecture. We classified the functional requirements (FRs) into software and hardware. The top six imperative FRs are transcribed below in Table 1 and Table 2.

Next, we provide more in-depth descriptions of the on-device components that make up the overall multimodal interaction functionality of the SA.

#### 2.2.1. Voice Management

**Figure 5:**
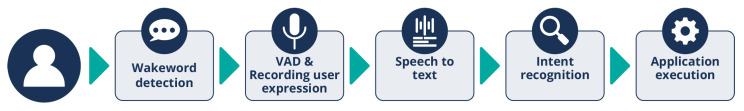
SA conversational interaction internal pipeline.

Figure 5 summarizes the conversational interaction functionality of the vocal interfaces. The user initiates the dialogue by stating a specific wake word. The assistant records the user’s command, converts it into text, and semantically processes it to determine the user’s intent and how the smart assistant should respond. Finally, depending on the user’s request, an application or device-handling command is executed and the SA provides the appropriate vocal response to the user.

In terms of the device’s speech services, two separate low-level API calls have been implemented. The first is the listen service, responsible for recording a sound from the microphone for a given amount of time and converting it to text, while the second is the speak service, responsible for converting a text to sound and playing it back on the speaker. The Google Cloud APIs were chosen due to their robustness, practicality, and multilingual ability for converting speech to text and vice versa.

To enhance the user experience of the voice interface, a Voice Activity Detection (VAD) service has been developed. VAD processes a sound stream in real time, and can determine whether someone is speaking; this allows the listen service to record sounds only when the user is talking (after the device has been enabled via the wake word detection) rather than recording for a fixed period of time, which could be either too long (the user would wait too long) or too short (the user might not manage to say all the words they wanted), both of which could lead to user frustration. Finally, in order for the VAD to work efficiently and recognize speech it needs to be pretrained to the environment’s noise level in quiet moments, a process that is carried out alongside wake word training.

#### 2.2.2. Wake Word Detection

A wake word is a special word or phrase that activates the device when spoken. In the context of an SA, it activates vocal interaction so that it is not constantly listening to the environment, which is both resource-intensive and privacy-invasive. The proposed SA uses Raven (https://github.com/rhasspy/rhasspy-wake-raven, accessed on 30 July 2024), an open-source wake word detector that can operate completely offline and handle multiple user profiles. Each profile requires three different template recordings of a given word or phrase. It also checks whether the recordings are clear enough or contain too much noise and whether they are similar in sound content. In addition, empirical tests have shown that phrases work better than words; thus, expressions such as “Hello Elsa” are preferred over a simple “Elsa” (in case the assistant was named *Elsa*), which can lead to several false positives. In addition, Raven performs real-time sound analysis using minimal resources and attempts to match a sound to one of these profile templates. The SA is equipped with a Wake Word System Manager responsible for handling all these processes, namely, running Raven, recording and storing the new Raven models during training, handling the different profiles, and notifying the device when a wake word is detected.

The training process is initiated from the graphical interface (touch screen) and the user is guided by vocal interaction with the SA. First, a few seconds of absolute silence are required to train the VAD. This is followed by three recordings in which the user provides the desired wake word. All templates are then postprocessed for noisy or clipped recordings, and any recordings of poor quality are repeated. Finally, the final template files are played back to the user for additional human review. In this way, each user can create, manage, and use their own personal wake word profile.

In conclusion, the wake word detection system is quite computationally efficient, allowing it to operate on the embedded Raspberry Pi 4 device with no issues (manufacturer is Raspberry Pi Foundation, Cambridge, United Kingdom).

#### 2.2.3. NLU Middleware

The core component of an SA is responsible for semantically processing each user utterance and deciding on the best response each time. The presented SA uses the Rasa framework for these tasks; as previously discussed, it offers the benefits of an open-source framework without sacrificing performance. Rasa consists of two modules: the Natural Language Understanding (NLU) module, responsible for intent recognition, and the Natural Language Generation (NLG) module, responsible for response selection and generation. Both of these require appropriate data for training. In particular, each dialogue scenario is an intent–response pair triggered by one or more different key phrases. These key phrases constitute the training data for each intent, while all pairs constitute the training data for the response selection system. For implementation of the NLU middleware, a Rasa management system has been developed that handles three processes: training a language model, querying an active language model, and supporting the conversation.

**Figure 6:**
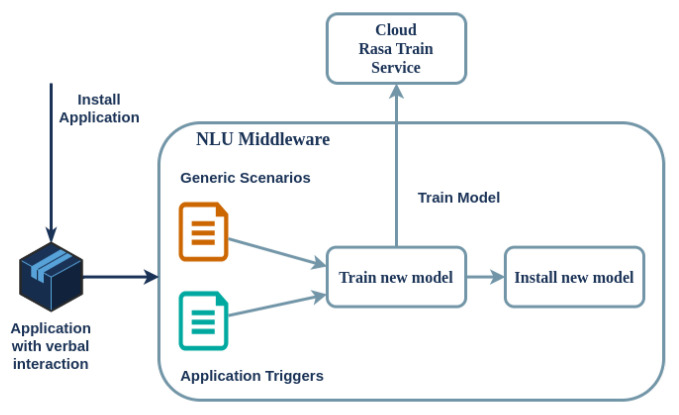
Decoupling training and inference of Rasa models for optimal performance of the SA on the edge.

More specifically, the dialogue scenarios are divided into two sections, namely, static and dynamic. Regarding the former, there are several generic and device-related scenarios, such as greeting, adjusting the volume, restarting the device, and specifying the installed applications. The latter are always present on the device, as they are defined in the NLU middleware; on the other hand, each uploaded multimodal application contains a set of trigger keywords (the intent examples) that are added (or deleted) each time an application is installed (or uninstalled) on a device. The NLU middleware collects all scenarios and associated data, creating necessary files for training when dynamic scenarios change. Training a Rasa model on the RPi-4 device can take up to half an hour, making it impractical to host on the edge. Therefore, a cloud-based training service has been implemented. The cloud service receives the training data and configuration parameters. After a brief period, typically 1–2 min, it sends the trained model back to the SA on the edge for deployment. The data sent to this cloud service contain only the intent examples from the *installed applications and the generic static conversations*, with no personal user data. This process is shown in Figure 6.

The NLU middleware also initiates and stops the existing model and manages the conversation; in particular, after a wake word is detected, it starts the conversation and uses the listen service (which internally uses the VAD functionality) to collect the user’s utterance. It then forwards the utterance to the Rasa model, which semantically understands and classifies it to the most relevant intent, then selects the appropriate response to send back to the manager. In order to handle these responses, a specific message format has been designed that describes whether it should initialize a specific application (and which one) or perform a device operation. It then completes the task by calling the appropriate device service via the on-device communication layer, implemented using Commlib internal DSL as described earlier.

#### 2.2.4. Haptic Interfaces

As discussed in Section 2.2, the SA is equipped with a touch screen, offering an alternative way of user interaction apart from the vocal modality. We opted for this solution, which diverges from the norm (as commercial SAs mainly support vocal interfaces), because our aim is to support several different target groups, such as the elderly, who often suffer from deteriorated hearing. Thus, it was important to provide an alternative and intuitive way of interacting with the device.

**Figure 7:**
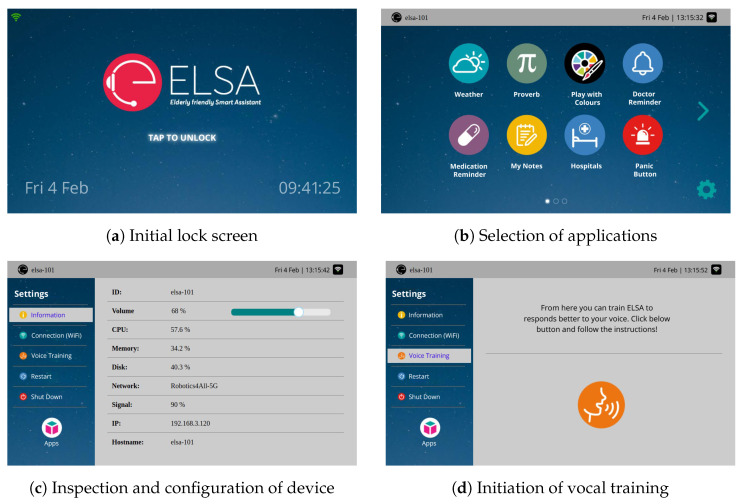
Static screens of the SA allowing for application initialization and device management.

Similar to vocal interfaces, haptic (touch) interfaces are split into static and dynamic. Static interfaces are essentially screens that allow for application deployment and device management (volume level, WiFi selection, vocal training, and adaptation, among others). Examples of such screens exist in Figure 7.

**Figure 8:**
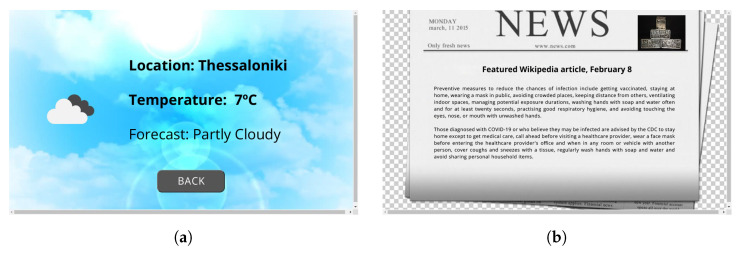
Dynamic screens of SA that are initiated and terminated along with the respective applications: (**a**) visual interface of the weather reporting application and (**b**) visual interface of the Wikipedia article-of-the-day application.

Each dynamic application can support visual/haptic interaction by developing a web interface that initiates along with the application, ultimately taking over the screen until the application terminates. Examples of dynamically executed interfaces that accompany applications are shown in Figure 8.

Both static and dynamic haptic interfaces utilize the Webdis (https://webd.is/, accessed on 30 July 2024) software to communicate with the SA’s local broker to receive information to visualize and dispatch haptic events to the system for management.

### 2.3. Application Management Methodology

In the context of the IoT and smart environments, remote installation and operation of applications on SA devices and robots is a common requirement. For instance, applications can be remotely installed on smartphone-type devices through the *Application Store*, which provides suitable interfaces for storing and downloading applications. In our case, the device connects to the *Application Store* remotely via the *Platform Broker*, from where it can query, download, and install an application locally on the device.

Listed below are the fundamental characteristics of the application management mechanism (*Application Manager*) that is installed and executed on SA devices:

- The mechanism implements application execution through container-based virtualization technologies, such as docker containers. It communicates with the local docker agent to manage application execution.
- The installed applications are stored in a local database, which is used to keep track of their state and status.
- It offers application management services, including application download, execution, and shutdown. It also provides asynchronous PubSub channels for sending debugging data and messages related to the current state of the application. Additionally, it generates event messages for internal application management functions, such as successful execution of the installation (*app_manager.events.app.installed*) and deletion (*app_manager.events.app.deleted*) processes.
- It connects to a message broker, which provides access to the application management services mentioned above. These services can be invoked from both the edge and cloud layers through the message broker.
- Identification messages are sent at regular intervals, including *heartbeat* identification packets that indicate the device’s active existence.
- A variety of applications are supported, including basic Python applications, vocal interaction applications, haptic interaction applications, and mixed interaction applications. The D-API and the C-API are used for interacting with the local resources (sensors, actuators, GUI Manager, and NLU middleware) and cloud services, respectively.
- The status of the application (logs, state, and health information) can be monitored in real-time.

The *Application Manager* is automatically deployed on the SA device at system startup, and provides services for executing, monitoring, and terminating applications. It interfaces with the rest of the system through either the local broker or the platform broker, enabling remote execution without the need to connect to the local network to which the device is connected.

**Figure 9:**
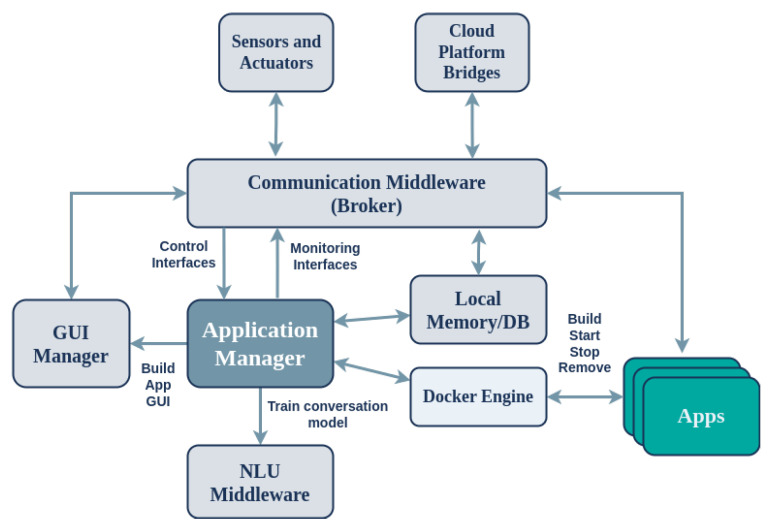
Architecture of the Application Manager running on the SA device. Arrows indicate communication actions.

As demonstrated in Figure 9, the Application Manager utilizes docker container technologies to execute applications in isolated environments. Additionally, it employs a local database (Redis DB) to store installed applications and their statuses. For remote application management and monitoring, it offers a series of interfaces (*Inbound Control Interfaces and Outbound Monitoring Interfaces*) to and from the platform via the *Platform Broker*.

The Application Manager provides several remote control and monitoring interfaces:

- **Control Interfaces:**
  – RPC service to start an application on the SA
  – RPC service to terminate a running application on the SA
  – RPC service to install an application on the SA
  – RPC service to execute an application for testing and debugging purposes without installing it
  – RPC service to check the aliveness of the Application Manager
  – RPC service to obtain the list of installed applications on the SA
  – RPC service to delete a previously installed application
  – RPC service to obtain the list of currently running applications on the SA
- **Monitoring Interfaces:**
  – Sends heartbeat messages; used to monitor the aliveness of the Application Manager
  – Sends a *started* event upon initialization of the Application Manager
  – Sends a *stopped* event upon termination of the Application Manager

In addition to the RPC interfaces mentioned above, the Application Manager generates and transmits messages in the form of *asynchronous events* to the message broker.

The *Application Manager* executes applications in docker container environments to isolate the application execution environment from the rest of the device system. In the context of the current study, we constructed corresponding docker images for four classes of applications: (a) Python applications that utilize the D-API and C-API; (b) Python applications with embedded vocal/conversational functionality; (c) Python applications with embedded haptic interaction functionality; and (c) Python applications for multi-modal haptic/vocal functionality. A multimodal interfaced application consists of the following files:

1. **app.py**: Executable Python application.
2. **requirements.txt**: Package/Module dependency definitions.
3. **init.conf**: Defines the initial variables of each application.
4. **app.info**: For compatibility purposes, the device’s name, version, and description are included in this file.
5. **exec.conf**: Incorporates information useful for scheduling and automating application executions.
6. **voice-commands.txt**: Defines the keyword(s) to be used by the NLU middleware for activating/starting the specific application via vocal commands.
7. **ui/main.html**: A directory named *ui* that contains at least one file named *main.html* offering visual/haptic capabilities to the application. This directory is optional.

Except for the *exec.conf* file, which is automatically generated, all files must be submitted from the developer to the application store compressed into a tarball file. For each application runtime, the appropriate interfaces are initiated to allow remote monitoring and management. In particular, the following endpoints are constructed by the *Application Manager* for each application execution:

- **Logging**: Sends application runtime logs to a topic.
- **Runtime Metrics (Stats)**: Sends application runtime metrics (e.g., CPU load, memory load, network usage) to a topic.
- **Application Started Event**: Sends an event to the relevant topic when an application is started.
- **Application Stopped Event**: Sends an event to the relevant topic when an application is stopped/terminated.

When dealing with applications that involve vocal and/or haptic (touch screen) interaction, the *Application Manager* communicates with the *NLU Middleware* and *GUI Manager* subsystems to create the necessary interfaces. In the case of vocal interaction, the conversational model is retrained on the cloud and then deployed back to the SA on the edge as previously described (refer to Figure 6). On the other hand, the appropriate GUI for the application is constructed, which defines both input and output interfaces for the touch screen of the SA. The application’s appropriate GUI is executed upon application startup via the *GUI Manager*, taking over SA’s touch screen. In this way, an application can support concurrently existing multimodal interfaces, as a user can give commands and at the same time select an item in the touch screen (provided that the application supports this functionality).

## 3. Demonstration Case Study

The presented architecture was instantiated in the SITIES project, which aims to create and deploy multimodal SA healthcare applications on the edge via implementation of *ELSA*, a Smart Assistant designed to provide senior citizens with autonomous living conditions and integrated health services. In this section, we present and discuss two case study scenarios of the ELSA SA: one for developing a conversational eHealth application, and a second for developing a haptic-based video call application for the device. The applications were deployed remotely via the provided web interface of the Application Store to the device on the edge.

**Figure 10:**
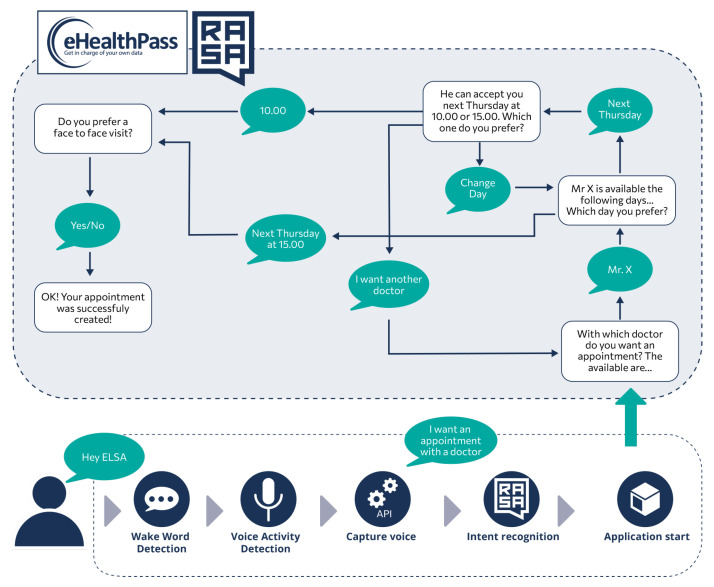
Activity of an interactive application example for scheduling health appointments via multimodal interfaces.

The end-to-end methodology for developing and deploying interactive applications can be better understood with an example use case, graphically depicted in Figure 10. Assuming that an ELSA user wants to handle her doctor appointments conversationally and already has an eHealthPass (https://www.gnomon.com.gr/ehealthpass/, accessed on 30 July 2024) account that connects with the doctor, she can install the developed “eHealthPass” ELSA application from the cloud platform and use it on demand. Particularly, patients or their relatives can connect to the cloud platform, select the application from the menu, and install it. Users should also declare the eHealthPass credentials that the application will use. The installation process takes place next. The cloud platform sends all data to the device, which first stores the Docker image of the newly added application and then prepares the voice commands for training. It merges the existing scenarios (the static scenarios and the already installed applications) with the new data and creates the appropriate Rasa configuration files, which are sent to the Rasa training cloud service for training. After a few minutes, training is finished and the received model is activated. The user is notified by a vocal message that the vocal commands are now ready for use.

After this procedure, users can execute the installed application verbally or via the touch screen, for example to request a new doctor’s visit (the lower part of Figure 10). More specifically, they must first activate the device by saying the wake word, which is usually “Hey ELSA”. The wake word detection module recognizes the particular phrase and informs the NPL middleware (Rasa) to start and handle a vocal conversation. In one case, blue LEDs are turned on to indicate that the SA is listening to user requests. Users can then say what they want to ensure that the most appropriate application is run. In this case, they should say “I want an appointment with a doctor” or a similar phrase, which is processed by the Rasa model after being converted to text via the D-API. The NLU middleware understands the user command and notifies the application manager to launch the most relevant application, in this case, eHealthPass.

In this particular application, a separate cloud-based eHealthPass-focused Rasa model has been trained to manage these conversations. The eHealthPass ELSA application is launched and the application manager passes the initial user request to the application, which is then sent to the remote Rasa model. This model understands and initializes the *request an appointment* scenario, which needs to collect the name of the doctor, the day and time of the visit, and its medium (face-to-face or remote) in order to submit a new doctor’s appointment. Each time, the Rasa model requests one of these fields by sending an appropriate message to the ELSA application, which dictates this message and listens to the user’s response. The application then converts the user’s utterance into text and sends it back to the remote Rasa system. When all the fields have been completed, the Rasa model sends the request to the eHealthPass platform and notifies the user that the process has been successfully completed and the application is closed.

**Figure 11:**
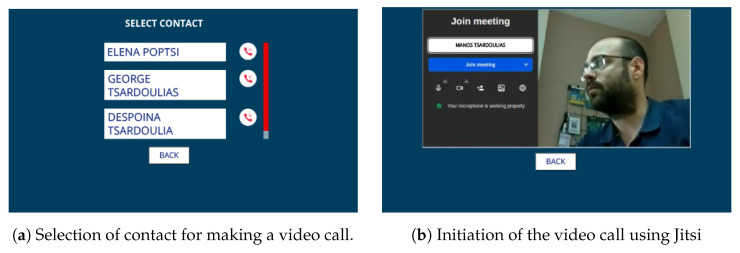
Haptic/visual interfaces of the video call application.

Following a similar process, ELSA users can install a video call application on their devices to connect with their friends and family via a Jitsi Meet (https://jitsi.org/, accessed on 30 July 2024) instance deployed exclusively for ELSA users (Figure 11). This application also considers that the users already have an eHealthPass account in which their friends’ contact info is stored. To initialize the app, users should say “I want to make a call” or a similar expression; when the Rasa model correctly understands the phrase, it orders the app to start. Then, a list of existing user contacts is presented on the screen and users touch the screen to select who they want to call. After that, a new Jitsi video call room is created and users are instantly connected using the microphone and camera of their ELSA devices. At the same time, their selected friends receive a text message containing a unique link to connect to the Jitsi room through their mobile phones. Finally, any time users want to exit the call, they can press the “Back” button, which terminates the call and returns the device to the main screen.

## 4. Discussion

In this work, we propose a multi-layer architecture that allows for remote distribution, management, and monitoring of multimodal applications on edge-based SA devices via the cloud. The proposed multilayered architecture consists of a cloud part, allowing for application submission and management, and device configuration; an edge SA device equipped with modular open-source software that allows for on-demand application management to support multimodal interaction; and a final connectivity layer consisting of local and remote brokers that glue all the components together. Although complex in its architecture, this approach allows for full modularization of all aspects of the system as well as extendability via additional services or IoT devices.

Regarding the hardware components, the choice of a Raspberry Pi 4 as the host for the SA can be deemed successful, although there is room for improvement. One significant limitation of the device is its reliance on an internet connection, which is a common drawback of most modern SAs. Although most components are hosted on the device, its ability to support complex and intelligent scenarios is limited. To avoid dependence on the local network, one solution could be to use 4G/5G services within the LAN on the edge to ensure high uptimes.

Furthermore, the conversational interface could be further improved. On the one hand, the wake word procedure of the SA could be refined, as users frequently faced challenges when trying to activate the device. On the other hand, enhancing the intelligence of local Rasa models to facilitate more intricate conversations on the edge may necessitate a more robust edge device such as a Jetson Nano. This could permit more sophisticated NLP models such as large language models to be hosted on Edge, allowing for multi-intent classification, entity recognition, complex scenario execution, and more natural and contextual interactions overall.

In closing, the proposed architecture offers several novelties. First, it avoids vendor lock-in, as it employs open software modules. The developed applications follow a small set of rules, promoting their integration into the app management system while allowing for maximum flexibility regarding their offered functionality. In addition, applications are deployed locally using containers, resulting in improved user privacy and isolation while securing user data against exposure to other applications or systems. Moreover, transparency is achieved, as the cloud platform shows the installed applications and verbal interactions of the SA in real time. Additionally, the proposed verbal interface and installed applications are selected and managed by the users, while applications can request personalized information from the user during application installation, resulting in a personalized SA. These aspects are significant, as they allow users to have complete internal knowledge and full ownership of the device they are using as well as the collected and processed data. Finally, we plan to conducting an extensive survey of end users and developers to evaluate aspects such as SA usability, user satisfaction and engagement, and ease of application development, with the aim of ultimately improving our architecture.

## Figures and Tables

**Table 1:** Functional requirements of the SA device relevant to the hardware.

ID	Description
FR1	The SA must provide vocal interfaces to interact with the user.
FR2	The SA must be able to understand vocal commands given by the user.
FR3	The SA must provide haptic interfaces to interact with the user.
FR4	The SA must be able to connect to WiFi networks.
FR5	The SA must be able to connect and interact with BLE devices.
FR6	The SA must provide speech and audio capabilities.

**Table 2:** Functional requirements of the SA device relevant to the software.

ID	Description
FR1	The SA must be able to internally and externally communicate with IoT-based message brokers.
FR2	The SA must include a speech-to-text module (ASR—Autonomous Speech Recognition), capable of interpreting verbal commands and associating them with the installed applications.
FR3	The SA must support applications management (downloading, installation, deployment, deletion).
FR4	The SA must include a user interface module, capable of showing information about the installed applications and the device’s status.
FR5	The SA must deploy an installed application in under 5 s.
FR6	The SA must correctly understand the user’s commands with a success percentage of over 80.

## Data Availability

No new data were created or analyzed in this study.

## Abbreviations

The following abbreviations are used in this manuscript:

SA	Smart Assistant
SAD	Smart Assistant Device
VA	Virtual Assistant
CPS	Cyber–Physical Systems
IoT	Internet of Things
